# Clinical, manometric, genetic, and histologic associations in pediatric intestinal pseudo‐obstruction: A case series

**DOI:** 10.1002/jpn3.70334

**Published:** 2026-01-01

**Authors:** Sharon Wolfson, Myra Butrviengpunt, Naomi Tjaden, Alain J. Benitez, Archana S. Kota, Jennifer Webster, Prasanna Kapavarapu, Hayat Mousa, Robert O. Heuckeroth

**Affiliations:** ^1^ Division of Pediatric Gastroenterology, Hepatology and Nutrition Children′s Hospital of Philadelphia Philadelphia Pennsylvania USA; ^2^ Department of Pediatrics, Perelman School of Medicine University of Pennsylvania Philadelphia Pennsylvania USA; ^3^ Touro College of Osteopathic Medicine Middletown New York USA; ^4^ Department of Pediatrics, Division of Pediatric Gastroenterology New York University Grossman School of Medicine New York New York USA

**Keywords:** ACTG2, chronic intestinal pseudo‐obstruction, intestinal myopathy, intestinal neuropathy

## Abstract

**Objectives:**

Pediatric intestinal pseudo‐obstruction (PIPO) is a severe bowel motility disorder characterized by impaired propulsion of gastrointestinal contents without mechanical obstruction. PIPO encompasses congenital and acquired disorders, including neuropathies, myopathies, and mesenchymopathies. PIPO presents with abdominal distension, bilious vomiting, and severe constipation. Diagnosis is based on objective measures of neuromuscular dysfunction, dilated bowel on imaging, parenteral and/or enteral nutrition dependence, and genetic or metabolic testing. Antroduodenal manometry permits objective assessment of proximal bowel neuromuscular function. Genetic testing is increasingly valuable although causes of PIPO often remain incompletely defined. Understanding genotype‐phenotype correlations is essential for clarifying disease mechanisms and guiding therapies. This study aimed to characterize the clinical and genetic profiles of children with PIPO, utilizing manometric data for subtype classification.

**Methods:**

A retrospective chart review was conducted at a tertiary care pediatric medical center, with inclusion criteria of PIPO diagnosis, completed manometry testing, and genetic evaluation.

**Results:**

Nineteen children met inclusion criteria. Antroduodenal manometry classified 59% as neuropathic, 35% as myopathic, and one with mixed neuropathic and myopathic dysfunction. Genetic testing revealed pathogenic *ACTG2* mutations in all myopathic cases, while neuropathic PIPO exhibited more genetic variability. Histopathology was inconsistent and often nonspecific. Therapeutic approaches focused on nutritional support and promotility agents, with surgical intervention more common in myopathic cases.

**Conclusions:**

This study highlights the association of *ACTG2* mutations with a myopathic phenotype, and genetic diversity in neuropathic PIPO, emphasizing the need for further research to improve phenotyping to enhance diagnosis and treatment.

## Abstract (highlights)

**What is Known:**

- Pediatric intestinal pseudo‐obstruction (PIPO) is a life‐threatening bowel motility disorder with significant morbidity.
- PIPO pathogenesis includes neuropathies, myopathies, and mesenchymopathies, with genetic testing facilitating some diagnoses.
- *ACTG2* mutations are the most common genetic cause of myopathic PIPO, characterized by distinct manometric patterns in bowel, bladder and uterine dysfunction, and often intestinal malrotation.

**What is New:**

- The data suggest genetic variability in neuropathic PIPO, with fewer known disease‐causing variants compared to myopathic PIPO.
- Histopathological findings in patients with PIPO are inconsistent and nonspecific.
- Successful therapeutic approaches for PIPO subtypes differ, with surgical interventions more common in myopathic cases.

## 1 INTRODUCTION

Coordinated bowel motility is essential for life. When bowel motility is profoundly abnormal, children become malnourished, have abdominal distension, vomiting, intractable constipation and may die without surgery, medication, or specialized nutrition strategies. Symptoms may mimic mechanical obstruction, but when dysmotility is the underlying problem and symptoms begin in childhood, the disease is called pediatric chronic intestinal pseudo‐obstruction (PIPO).

PIPO can stem from issues with smooth muscle, the enteric nervous system, interstitial cells of Cajal (ICC), PGDFRα+ cells, enteroendocrine cells, extrinsic bowel innervation, or the hypothalamic‐pituitary‐adrenal axis. Clinicians typically classify PIPO as “neuropathic” (visceral neuropathy), “myopathic” (visceral myopathy), “mixed,” or ICC‐related.^1^

With an estimated incidence of 1 in 40,000, PIPO is a serious condition; up to 30% of affected children die, and many require lifelong nutritional support and frequent hospitalizations.^2^, ^3^, ^4^ Diagnosis requires at least two of the following: objective evidence of small intestinal neuromuscular dysfunction (via manometry, histopathology, or transit studies), recurrent and/or persistently dilated loops of small intestine with air fluid levels identified on X‐ray, dependence on enteral (i.e., tube feeding) or parenteral nutrition due to feeding intolerance at least intermittently or identified genetic mutations and/or metabolic abnormalities known to cause PIPO.

Genetic testing is increasingly used, revealing a growing list of gene mutations associated with PIPO. However, many cases remain idiopathic.^5^ Understanding genotype‐phenotype correlations is vital for clarifying disease mechanisms and guiding targeted therapies.^5^, ^6^ This study aims to characterize the clinical and genetic profiles of children with PIPO, leveraging manometric findings to classify subtypes in relation to genetic testing results.

## 2 METHODS

A retrospective chart review of pediatric patients was conducted at Children′s Hospital of Philadelphia (CHOP), a tertiary care center, from January 1, 2012 to November 1, 2023. An Epic electronic health record query was used to identify eligible patients. De‐identified data were securely stored and managed on Excel.

### 2.1 Ethics statement

The study was approved by the Institutional Review Board at CHOP.

### 2.2 Patient selection rationale

The symptoms of PIPO can vary from person to person, with a spectrum of clinical phenotypes ranging in severity.^7^, ^8^ While some people with PIPO experience continuous, severe symptoms requiring intensive nutritional and surgical support, others may have fluctuating disease courses with periods of relative stability.^4^, ^7^ Modern management strategies including optimized enteral nutrition and prokinetic medications may allow some patients to achieve periods of improved enteral and oral nutritional tolerance.^9^, ^10^ We intended to capture the clinical heterogeneity of this cohort through the following inclusion and exclusion criteria and electronic medical record subject identification strategy. This approach aligns with current clinical guidelines,^7^ enabling the examination of genotype‐phenotype relationships across the full disease spectrum that range from milder clinical presentations to intestinal failure.

#### 2.2.1 Inclusion criteria

(1) Diagnosis of PIPO. (2) Completion of antroduodenal or colon manometry testing with raw tracings available. (3) Evaluation by Genetics Division/Department with completion and review of recommended genetic testing. We allowed for inclusion of different types of genetic testing as recommended for clinical evaluation including: targeted gene panels, SNP arrays, whole exome sequencing, whole genome sequencing and mitochondrial DNA analysis.

#### 2.2.2 Exclusion criteria

(1) Lack of manometry tracings. (2) Uncertainty about PIPO diagnosis (e.g., clinical suspicion for PIPO, but normal ADM).

### 2.3 Subject identification

#### 2.3.1 Diagnosis and procedure codes used in Epic medical record search

International Classification of Diseases (ICD) 9 or 10 codes: ICD‐10‐Z15.89 (monoallelic mutation of the *ACTG2* gene); ICD‐9‐CM 560.89 and ICD‐10‐K58.89 (intestinal pseudo‐obstruction), and Current Procedural Terminology (CPT) codes 91020, 91022 (ADM), 91117 (colon manometry), and 91122 (anorectal manometry).

#### 2.3.2 Chart review

Identified charts were independently reviewed by two authors (a medical student and an advanced fellow in pediatric neurogastroenterology and motility). Discrepancies were resolved by discussion, with final adjudication by a pediatric neurogastroenterology supervising attending. Extracted data include demographics, medical and surgical history, presenting and ongoing symptoms, pharmacologic and nutritional interventions, imaging and laboratory results, manometry data and genetic evaluation. Genetic evaluation included extraction of genetic test reports along with interpretation of these reports documented in genetic clinical notes.

### 2.4 Antroduodenal manometry (ADM)

ADM testing was completed as part of clinical diagnostic evaluation for PIPO as per our institutional protocol. A water‐perfused manometry catheter with recording ports was placed by an interventional radiologist. The ADM catheter was positioned to ensure at least 2–3 of the distal recording ports in the small intestine beyond the ligament of Treitz and 1–2 of the most proximal ports in the gastric antrum. Following placement of this catheter and patient recovery from sedation, recording was started and included a 2–3 h fasting period followed by intravenous administration of erythromycin 1 mg/kg or azithromycin 1 mg/kg with a subsequent 1‐h monitoring period. If inadequate response was seen, an optional 3 mg/kg dose of erythromycin was given. This was followed by a standardized test meal (via mouth or gastric enteral bolus) with 2‐h post‐prandial observation. In some cases, attending neurogastroenterology physicians also gave sub‐cutaneous octreotide 1 μg/kg followed by 30–60 min of additional monitoring.

Manometry tracings were analyzed using Laborie software (version 10.0, build 2902) by an advanced fellow under the supervision of an attending physician with expertise in neurogastroenterology and motility. Interpretation followed guidelines from the North American Society for Pediatric Gastroenterology, Hepatology and Nutrition (NASPGHAN) and The American Neurogastroenterology and Motility Society (ANMS).^7^, ^11^, ^12^, ^13^ “Normal” ADM was defined by the presence of coordinated, propagating Phase III migrating motor complexes (MMCs) during fasting or following erythromycin or octreotide administration, and the presence of a postprandial conversion to a fed pattern. Myopathic PIPO was characterized by low‐amplitude contractions (typically <20 mmHg) with preserved propagation and coordination, indicating impaired smooth muscle function. Neuropathic PIPO was defined by the presence of any of these abnormal findings: absence of MMCs during fasting and following erythromycin (or azithromycin) administration, or abnormal migration or disorganized Phase III MMC, or presence of sustained tonic phasic contractions, or failure to transition to a fed pattern following meal challenge (postprandial hypomotility).^11^, ^12^, ^13^, ^14^

### 2.5 Statistical analysis

Descriptive findings are summarized without statistical analyses given the small sample size. Figures were prepared with GraphPad Prism 10 (GraphPad Software 10.1.0 (316)) and Adobe Illustrator 27.9.6.

## 3 RESULTS

### 3.1 Patient overview

**Figure 1:**
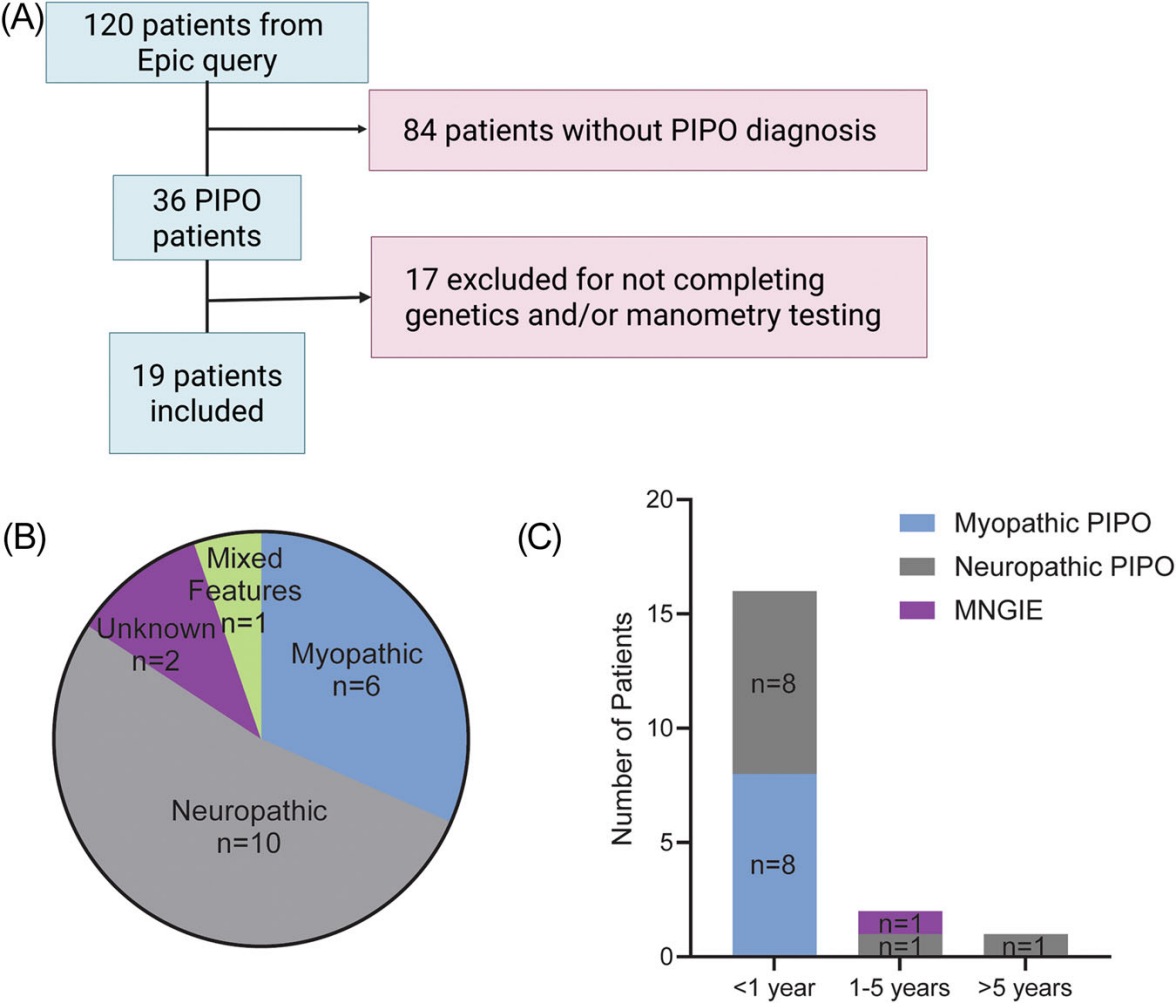
Study design, cohort subtyping by ADM and age of symptom onset. (A) Study design and inclusion/exclusion criteria. (B) PIPO subtype by ADM. There were 10 children with neuropathic PIPO (gray), 6 children with myopathic PIPO (blue), 2 children with unknown ADM results (purple, these patients only completed colonic manometry [both of which showed absence of any high amplitude propagating contractions]), 1 with both myopathic and neuropathic ADM findings (green). (C) Age of onset by PIPO subtype. Myopathic patients all presented prior to 1 year of age (blue), neuropathic patients presented at all age categories (gray), and one MNGIE patient identified by genetic testing (purple) presented at age. (3). This patient did not have ADM testing. ADM, antroduodenal manometry; MNGIE, mitochondrial neurogastrointestinal encephalomyopathy; PIPO, pediatric intestinal pseudo‐obstruction.

Our Epic query identified 120 individuals potentially having PIPO based on ICD and CPT codes. Of these, 84 were excluded as PIPO was not their final diagnosis. From the remaining charts, 36 had a clinical diagnosis of PIPO,^7^ but 17 were excluded because of incomplete genetic testing or unavailable manometry tracings. Ultimately, 19 children met all inclusion criteria and were analyzed in the final cohort (Figure 1A and Table 1).

**Table 1:** Clinical characteristics and treatments in PIPO cohort.

Pt ID	Age at symptom onset	Age at PIPO diagnosis	Genetic diagnosis (amino‐acid and/or DNA change)	ADM diagnosis	ADM abnormality	Colon manometry results	Pathology findings	Other congenital abnormalities	Intestinal diversion (type)	G‐tube/J‐tube	Nutrition source	Stopped promotility agents	Current promotility agents
1	2 months	10 months	*ACTG2 (p.R257C)*	Myopathy	Low amplitude intestinal contractions	Not applicable (NA)	Ileostomy: unremarkable	Malrotation, Dilated bladder	Yes – ileostomy (later multivisceral transplant)	Yes	Total parenteral nutrition (TPN)	Erythromycin (ineffective)	None
2	6 months	6 months	*ACTG2 (p.E196D)*	Myopathy	Low amplitude intestinal contractions	No high amplitude propagating contractions (HAPCs), low amplitude propagating contractions (LAPCs) present	NA	Malrotation, dilated bladder	Yes – ileostomy	Yes	Enteral nutrition (EN) + PN	Erythr omycin pyridostigmine prucalopride cisapride (i neffective)	Metoclopramide, augmentin
3	0–2 months	11 years	*ACTG2 (p.T195I)*	Myopathy	Low amplitude intestinal contractions	No HAPCs, No LAPCs	Ileum: unremarkable	Malrotation	Yes – transverse colostomy	Yes	Oral + EN	Pyridostigmine (ineffective)	Prucalopride, linaclotide, cecostomy flushes
4	1 week	3 years	*ACTG2 (p.Y144F)*	Myopathy	Low amplitude intestinal contractions	No HAPCs, No LAPCs	Colon: patchy hypertrophy, mild fibrosis	Dilated bladder	No	No	Oral	NA	Erythromycin
5	Birth	4 months	*ACTG2 (p.R257C)*	Myopathy	Low amplitude intestinal contractions	No HAPCs, No LAPCs	Jejunum: ↓ SMA staining, otherwise unremarkable	Malrotation, Dilated bladder	Yes – ileostomy	Yes	EN + PN	Pyridostigmine (high stoma output)	—
6	Birth	11 years	*ACTG2 (p.R257C)*	Myopathy	Low amplitude intestinal contractions	No HAPCs, LAPCs present	NA	Congenital hydronephrosis, Malrotation	No	No	Oral	NA	Erythromycin, pyridostigmine
7	Birth	1 month	*ACTG2 (p.R257C)*	NA	NA	No HAPCs, No LAPCs	Ileostomy: unremarkable	Dilated bladder	Yes – ileostomy	Yes	EN + PN	NA	—
8	3 months	13 years	*ACTG2 (p.R257H)*	Myopathy & Neuropathy	Disorganized phase 3 migrating motor complex (MMC), low pyloric amplitude	No HAPCs, LAPCs present	Rectum: unremarkable	None	No	Yes	EN + PN	Erythromycin (ineffective), linaclotide (high stool output)	Pyridostigmine, prucalopride, augmentin
9	2 years	2 years	*None detected*	Neuropathy	Disorganized phase 3 MMC	No HAPCs, No LAPCs	NA	Congenital unilateral hearing loss	No	No	Oral	—	Linaclotide
10	Birth	Birth	*Chrom 2q32.3 dup, 14q21.2 del, GUCY2C*	Neuropathy	Absent phase 3 MMC, simultaneous contractions	No HAPCs, No LAPCs	Ileum, colon, rectum: unremarkable	None	Yes – ileostomy	Yes	EN + PN	Erythromycin (ineffective)	Cisapride, augmentin
11	Birth	3 months	*COL3A1 mutation c.2002C>A (p.P668T)* *LDB3 c.1535 A* > *C (p.Q512P)* *VKORC1 (heterozygous c.1639 G* > *A)* *CYP2D6 (c.506‐1 G* > A, CYP2D6*4)	Neuropathy	Disorganized phase 3 MMC	No HAPCs, No LAPCs	Rectum: unremarkable	None	No	Yes	EN + PN	Cisapride, pyridostigminemetoclopramide, erythromycin (ineffective)	Prucalopride
12	Birth	5 months	*RET ([c.337+9 G* > *GA; c.1296 A* > *G (p.A432A); c.2307 T* > *TG (p.L769L)* + *L12)*	Neuropathy	Disorganized phase 3 MMC, pressurization	No HAPCs, No LAPCs	↓ Ganglion cells, absent calretinin	None	Yes – ileostomy	Yes	TPN	Metoclopramide, erythromycin (ineffective); pyridostigmine (high stoma output)	Augmentin
13	Infancy	<1 year	*EDNRB* *(het for c.821 A* > *G; p.D274G)*	Neuropathy	Disorganized phase 3 MMC	HAPCs	Colon: Unremarkable	None	No	Yes	EN	—	—
14	Birth	1 year	*PMM2 (c.422 G* > *A;p.Arg141His) (Heterozygous)* *KMT2A (c.5165 A* > *G; p.N1722S) (Heterogeneous)*	Neuropathy	Disorganized phase 3 MMC	HAPCs	NA	Autism, tethered cord	No	Yes	EN	Cisapride (effective but inaccessible); erythromycin, metoclopramide (ineffective)	Prucalopride
15	10 years	16 years	*MT‐ATP‐6* *(homoplasmic VUS in ATP6, 8843 T* > *C (p.I106T, ATP6))*	Neuropathy	Absent fed response	HAPCs to ascending colon	Colon/ileum: unremarkable	Autonomic dysfunction, hemophagocytic lymphohistiocytosis	Yes – ileostomy	Yes	TPN	Metoclopramide, erythromycin, pyridostigmine (ineffective)	Prucalopride
16	Prenatal	2 days	*FLG mutation (c.7339 C* > *Tp. (p.R2447))*	Neuropathy	High pyloric amp., no octreotide response	No HAPCs, No LAPCs	Ileum: congenital hypoganglionosis	None	No	Yes	EN + PN	Erythromycin (ineffective)	Pyridostigmine, prucalopride, augmentin
17	Birth	3 years	*EYA4 mut., Chr1 partial del (1q21.1q21.2)*	Neuropathy	Disorganized phase 3 MMC, antral hypomotility	HAPCs to sigmoid	NA	Multicystic dysplastic kidneys	No, cecostomy	Yes	Oral + EN	Augmentin (ineffective), prucalopride (fatigue/irritability)	Pyridostigmine, augmentin
18	Birth	Birth	*POLG variant (c.2221 G* > *A, p.D741N)*	Neuropathy	Non‐propagated bursts, no organized MMC	No HAPCs, LAPCs present	NA	CMV enteritis, developmental delay	No	No	TPN	Metoclopramide, erythromycin (ineffective)	Augmentin
19	3 years	3 years	*MNGIE (LIG3, c. 2890 G* > *C, p.G964R)*	NA	—	No HAPCs, No LAPCs	Colon: severe muscular atrophy, fibrosis, submucosal fat	Cerebral palsy, urinary retention, vesicoureteral reflux	Yes – ileostomy	Yes	TPN	—	—

Abbreviations: ADM, antroduodenal manometry; EN, enteral nutrition; HAPCs, high amplitude propagating contractions; LAPCs, low amplitude propagating contractions; MMC, migrating motor complex; PIPO, pediatric intestinal pseudo‐obstruction; SMA, smooth muscle alpha; TPN, total parenteral nutrition.

### 3.2 ADM results and clinical presentation

ADM was completed in 17 of 19 patients and used to determine PIPO subtype. 59% (*n* = 10) were classified as neuropathic PIPO, 35% (*n* = 6) as myopathic PIPO, and one patient exhibited mixed features (Figure 1B). Two patients completed colonic manometry alone, precluding precise PIPO classification (Figure 1B, “unknown”), but showed absent high amplitude propagating contractions (HAPC) postmeal and bisacodyl stimulation, consistent with colonic inertia.

Symptom onset occurred within the first year of life for 16 of 19 children (Figure 1C). Presenting symptoms included abdominal distension, enteral feeding intolerance, vomiting, bilious emesis, and severe constipation. Feeding intolerance was defined by clinical notes describing inability to advance feeds due to severe distension, emesis or gastric drainage of feeds. Intestinal malrotation, urinary tract abnormalities, and neurodevelopmental disorders were identified in several patients (Table 1). Two children with neuropathic PIPO had symptom onset at 2 and 10 years of age (Figure 1C). One child developed symptoms at age 3 and was later diagnosed with mitochondrial neurogastrointestinal encephalomyopathy (MNGIE) (Figure 1C).

**Figure 2:**
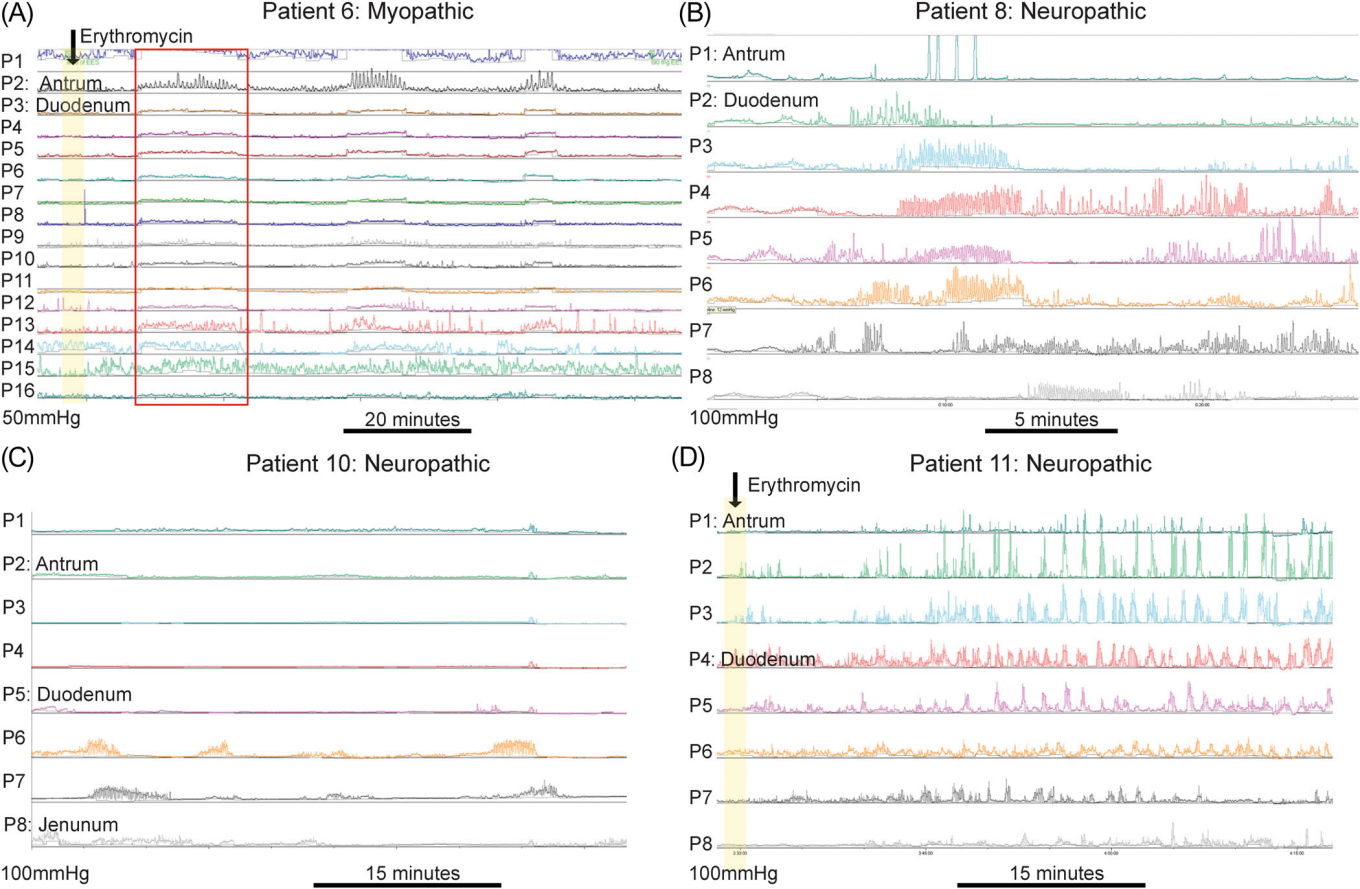
Example ADM findings in PIPO cohort. Analysis was completed via use of ANMS and NASPGHAN guidelines. (A) Patient 6 myopathic changes with coordinated but low amplitude (<30 mmHg) contractions (red box) following erythromycin administration (yellow overlay and arrow). Scale bar indicates 20 min. (B) Patient 8 fasting state tracing with disorganized MMC and evidence of retrograde activity. Scale bar indicates 5 min. (C) Patient 10 with neuropathic ADM findings with duodenal and jejunal bursts in the fed state, without antral activity (red boxes). Scale bar indicates 15 min. (D) Patient 11 with neuropathic findings with IV erythromycin triggering phasic small bowel contractions with amplitude >30 mmHg which did not propagate into the small bowel. Duodenal tracing showed regular non‐propagating clusters of intestinal pressure combining phasic and slight tonic activity. Jejunal activity remained relatively quiescent. Scale bar indicates 15 min. ADM, antroduodenal manometry; ANMS, American Neurogastroenterology and Motility Society; MMC, migrating motor complex; NASPGHAN, North American Society for Pediatric Gastroenterology, Hepatology and Nutrition; PIPO, pediatric intestinal pseudo‐obstruction.

Representative ADM tracings are in Figure 2. Myopathic PIPO was diagnosed by low‐amplitude but coordinated small bowel contractions (Figure 2A). Neuropathic PIPO showed disorganized motility patterns, including retrograde Phase 3 MMCs, segmental bursts of activity/pressurization, absent fed response, or impaired anterograde propagation (Figure 2B–D).

### 3.3 Genetics

Genetic variants were identified on clinical testing in 18 of 19 children with PIPO. Twelve variants were considered potentially related (Table 1).

All six children diagnosed with myopathic PIPO had pathogenic heterozygous missense mutations in *ACTG2*. An additional child with “mixed” features on ADM and one child who did not complete ADM also had heterozygous pathogenic *ACTG2* variants, classifying them as myopathic PIPO based on genetics. Five distinct *ACTG2* missense mutations were identified, with *ACTG2 R257C* present in five of eight affected children (Table 1).

For neuropathic PIPO diagnosed by ADM, 9 of 10 had identified pathologic gene variants, though only three variants were considered PIPO‐related (*EDNRB (Endothelin receptor type B)*, *RET (RET protooncogene)*, and *mitochondrial MT‐ATP6 variant m.8843 T*). Additional children with neuropathic PIPO had variants in *FLG* (*Filaggrin*), *COL3A1* (*Collagen type III alpha 1 chain*), *KMT2A* (*Lysine methyltransferase 2A*), and *EYA4* (*EYA transcriptional coactivator and phosphatase 4*) (Table 1). One child with neuropathic PIPO had a *GUCY2C* (*Guanylate cyclase 2C*) variant accompanied by a 2q32.3 duplication and 14q21.2 deletion. Another child with neuropathic PIPO had a variant of unknown significance in *POLG* (*DNA polymerase gamma, catalytic subunit*) (carrier status), but this child also had a history of congenital cytomegalovirus (CMV) enteritis complicated by intestinal failure as an infant. Finally, one child was diagnosed with mitochondrial neurogastrointestinal encephalomyopathy (MNGIE) due to a *LIG3* (*DNA ligase 3*) mutation but was not evaluated by manometry.

### 3.4 Histopathology linked to genotype

Histopathology reports from full‐thickness bowel biopsies were available in 13 of 19 subjects. Specimens were obtained from jejunum, ileum, colon and rectum. Abnormal findings were reported in 5 of 13 subjects.

Six of eight children with ACTG2 mutations had full thickness biopsies. Four of these appeared normal. One child with an *ACTG2 Y144F* variant showed patchy smooth muscle hypertrophy, mild fibrosis and a few intranuclear vacuoles in muscularis propria of the colon, plus patchy submucosal edema. One child with an *ACTG2 R257C* variant had decreased smooth muscle alpha (SMA) immunostaining in the circular layers of the muscularis propria of the jejunum, but otherwise normal histology.

Six of 10 children with neuropathic PIPO had available full‐thickness biopsies. The child with a *RET* mutation had sparse ganglion cells in the myenteric plexus with absent calretinin staining in the ileum and colon, as well as thinned, fibrotic and irregular outer longitudinal muscularis propria in the colon. The child with an *FLG* mutation had myenteric hypoganglionosis in ileal tissue. The child with a *LIG3* mutation had marked thinning and fibrosis of the muscularis propria.

### 3.5 Clinical phenotype linked to genotype

Clinical phenotypes are summarized in Table 1. Five of 8 children with *ACTG2* mutations had intestinal malrotation, a finding unique to this group. Five children with *ACTG2* mutations also had bladder distension, and one had congenital hydronephrosis. The child with a *LIG3* mutation had cerebral palsy, urinary retention, vesicoureteral reflux, and was completely unresponsive to promotility therapies. The child with an *EYA4* mutation had multicystic dysplastic kidneys. The child with a *KMT2A* mutation had autism and tethered cord. The child with the *MT‐ATP‐6 m.8843 T* mutation exhibited autonomic dysfunction and hemophagocytic lymphohistiocytosis. The child with no identified pathogenic gene variants has congenital unilateral hearing loss.

### 3.6 Clinical course linked to genotype

**Figure 3:**
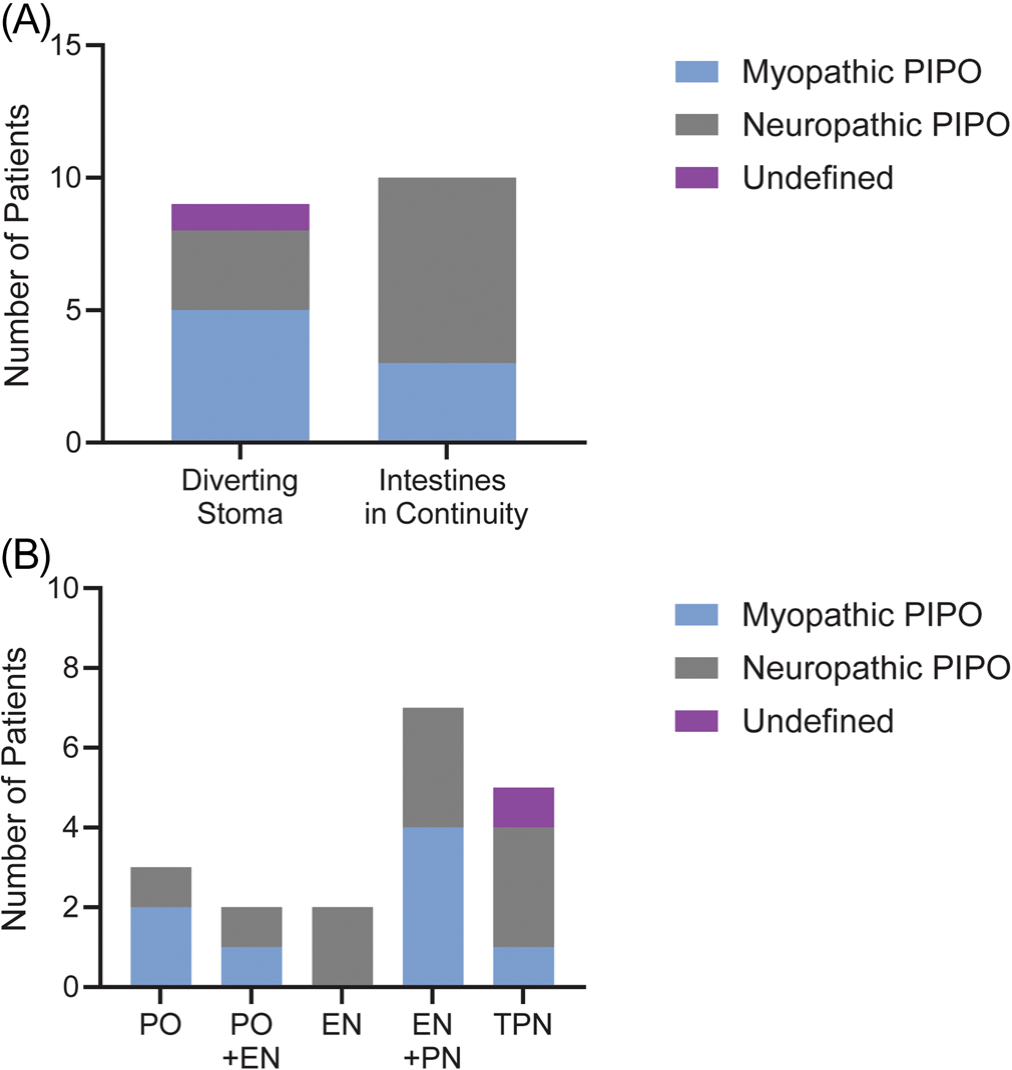
Diversion and nutritional requirements. (A) Bar graphs indicate numbers of patients with a diverting stoma versus individuals with intestines in continuity. (B) Bar graphs indicate numbers of patients with distinct types of nutritional support. EN, enteral nutrition; PIPO, pediatric intestinal pseudo‐obstruction; PN, parenteral nutrition; PO, oral feeding; TPN, total parenteral nutrition.

At last follow up, ages ranged from 2 to 30 years old. One subject died, unrelated to PIPO. For *ACTG2*‐related myopathic PIPO, five of eight (62.5%) underwent surgical diversion (four ileostomies, one transverse colostomy), primarily for decompression (Figure 3A). One child with *ACTG2 R257C* mutation had an ileostomy at 1 years of age subsequently underwent small bowel and large bowel transplant at 15 years of age. Another child with *ACTG2 T159I* mutation had a transverse colostomy and a cecostomy to aid with stool evacuation. In contrast, only three children with neuropathic PIPO had ileostomies (30%), and one additional child with neuropathic PIPO underwent cecostomy to manage refractory constipation (Figure 3A).

Nutritional support was required across both PIPO subtypes, with most dependent on enteral and/or parenteral nutrition (PN) (Figure 3B). Five individuals were fully PN‐dependent.

Nearly all subjects received pro‐motility medications. Common therapies included serotonin receptor type 4 (5‐HTR4) agonists (prucalopride and cisapride), cholinesterase inhibitors (pyridostigmine), motilin‐receptor agonists (erythromycin and azithromycin), dopamine receptor (D2) antagonist/5‐HTR4 agonist (metoclopramide), and pro‐motility antibiotics (amoxicillin‐clavulanate). Cisapride improved symptoms in one neuropathic PIPO patient but was not available for continued use. Erythromycin and metoclopramide were generally ineffective. Pyridostigmine was stopped in two subjects due to high stool output (Table 1).

## 4 DISCUSSION

PIPO represents the most severe pediatric motility disorder,^8^ yet encompasses a spectrum of disease severity rather than one uniform phenotype. Affected children commonly suffer from massive abdominal distension, vomiting, intractable constipation, and require at least partial intravenous nutrition. However, disease severity may fluctuate over time and modern management strategies aim to optimize enteral tolerance whenever possible.^10^ Our cohort reflects this clinical heterogeneity, ranging from patients requiring continuous parenteral nutrition and surgical decompression to those achieving periods of relative stability with optimized medical management.

Our cohort fulfilled PIPO diagnostic criteria through the combination of expert clinical evaluations, and manometric and genetic testing results.^7^ All patients demonstrated pathognomonic ADM findings‐ absent or abnormal MMCs, failure to achieve fed patterns, or low‐amplitude contractions‐distinguishing true intestinal dysmotility from other disorders of brain gut interaction.

The causes of PIPO remain incompletely defined, especially for neuropathic forms. In contrast, most children with myopathic PIPO have disease‐causing mutations in genes critical for actin‐myosin interactions. In our cohort, all the children with visceral myopathy had heterozygous *ACTG2* point mutations, which account for 44% of previously reported myopathic PIPO‐inducing mutations.^6^ While other genes like myosin heavy chain 11 (*MYH11*), myosin light chain 9 (*MYK9*), myosin light chain kinase (*MYLK*), Leiomodin 1 (*LMOD1*), Filamin A (*FLNA*), and specific mutations in alpha smooth muscle actin (*ACTA2 R179H or ACTA2 R179C*) can also cause myopathic PIPO, our findings emphasize ADM′s utility in subtyping PIPO and confirm a strong association between *ACTG2* mutations and myopathic manometric patterns. Interestingly one child whose manometry showed “mixed” (myopathic and neuropathic) features also had a pathogenic *ACTG2 R257H* mutation, suggesting other factors like surgical history might influence these findings. As previously reported, pathogenic *ACTG2* mutations commonly cause bladder dysfunction; six of eight in our cohort had identified bladder dysfunction.

Phenotypic variability among patients with identical *ACTG2* mutations was striking. While all children with the *ACTG2 R257C* mutation had enlarged bladders, only three of four had malrotation, and only two developed midgut volvulus. None had microcolon, although microcolon is commonly reported. One person with this mutation experienced a milder course, requiring only medical management without need for ostomy or gastrostomy. Phenotypic diversity also existed in individuals with different *ACTG2* mutations.

This phenotypic diversity, even among people with identical*ACTG2* mutations, has been previously reported^6^, ^15^, ^16^ and raises questions about whether specific *ACTG2* mutations differentially impact intestinal smooth muscle function. These findings suggest second‐site genetic modifiers, environmental and/or epigenetic factors play a role in varying clinical presentations^17^, ^18^ and highlight that PIPO diagnosis should rely on objective manometric and genetic findings rather than clinical severity alone, which may fluctuate substantially. Our results support prior studies identifying *ACTG2* mutations as a leading monogenic cause of PIPO, reinforcing that *ACTG2*‐related disease represents a distinct clinicopathologic entity.^15^, ^17^, ^19^

In contrast, the neuropathic PIPO subgroup exhibited more variable manometric patterns and genetic findings. Although potentially pathogenic genetic variants were identified in nearly all patients with neuropathic ADM findings, only a minority were considered likely PIPO‐associated. Variants in *RET* and *EDNRB* were present in two people; inactivating mutations in these genes cause Hirschsprung disease, a disorder where the enteric nervous system is absent from distal bowel.^20^ The child with the*RET* variants had histopathologic evidence of hypoganglionosis, which also occurs in mice with reduced *RET* signaling.^21^ However, other variants (*COL3A1*, *FLG*, and *GUCY2C*) lacked known pathogenicity, possibly representing incidental findings or genetic modifiers. One patient had a single autosomal recessive *POLG* variant. While some *POLG* variants are associated with MNGIE, this variant was not deemed to be pathogenic by our geneticists, and this patient′s congenital CMV enteritis was considered a more likely etiology for intestinal neuropathy. These observations underscore challenges in interpreting next‐generation sequencing data in rare disease populations.

Histopathologic data, while valuable in some cases, was often non‐diagnostic. For most people with *ACTG2* mutations, histology was unremarkable or findings were nonspecific despite clear manometric and clinical evidence of myopathy, consistent with prior studies.^22^ One child with*ACTG2 Y144F* showed hypertrophy and mild fibrosis of the colonic muscularis propria, while one with *ACTG2 R257C* had decreased alpha smooth muscle immunostaining. These observations suggest we need to apply new analytic strategies to identify correlates of dysmotility in biopsies. These new strategies might include tissue clearing, multi‐label immunohistochemistry and confocal imaging,^23^, ^24^ spatial transcriptomics, or RNA sequencing. RNA analyses might be particularly valuable because smooth muscle undergo remarkable changes in gene expression and function in response to various stressors.^25^, ^26^ This plasticity in smooth muscle is well‐documented in the vasculature and called “phenotypic class switching,” a change accompanied by marked reductions in contractile gene expression that could explain reduced alpha smooth muscle actin in our patient.^27^ If this occurs in myopathic PIPO, then alternative strategies could target this process to enhance contractile smooth muscle phenotype.

Neuropathic PIPO histopathology was also mostly unremarkable, except for hypoganglionosis in children with *RET* or *FLG* variants. We speculate that expanding histologic examination of full‐thickness biopsies to include three‐dimensional imaging after tissue clearing may offer further diagnostic utility in neuropathic PIPO since neuron counts are more reliable in three‐dimensions and enteric nervous system organization is difficult to appreciate in thin sections.^23^, ^24^ Full‐thickness intestinal samples may also identify inflammatory causes of chronic intestinal pseudo‐obstruction.^28^

Therapeutic approaches were similar across subtypes, and centered around intestinal decompression (ostomy), nutritional rehabilitation (intravenous or enteral), and pro‐motility medicines, consistent with recommended guidelines and previously published treatment strategies.^7^, ^10^, ^29^, ^30^, ^31^, ^32^, ^33^, ^34^ No single promotility agent demonstrated consistent efficacy, highlighting the urgent need for targeted, mechanism‐based treatments.

This study has several limitations. The small cohort size and the retrospective design limit generalizability. Our cohort includes patients with varying clinical severity, reflecting PIPO′s heterogeneous nature as described in consensus guidelines. While all patients met established diagnostic criteria, inclusion across the severity spectrum may complicate direct comparisons with studies focused exclusively on the most severe phenotypes. Nevertheless, this inclusive approach enhances generalizability and reflects real‐world practice where disease severity fluctuates. Heterogeneity in testing (genetic panels, histologic sampling locations) is an additional limitation. Since ADM testing at our center occurs on the day of catheter placement, potential sedation effects exist,^35^ though our protocol consistently waits for full anesthesia recovery before recording. Lastly, treatment decisions were made by numerous clinicians often across multiple institutions and reported approaches are not intended as treatment guidelines.

## 5 CONCLUSIONS

Our study offers insight into the complex landscape of PIPO, a devastating motility disorder. Our findings strongly reinforce the well‐established association of *ACTG2* mutations with a distinctive myopathic phenotype, underscoring its emerging recognition as a specific clinicopathologic entity within the broader, more heterogeneous spectrum of PIPO.

In stark contrast, our data highlight the persistent difficulty in defining the underlying causes of neuropathic PIPO using currently available genetic, manometric, and histopathologic analyses. While genetic variants were identified in nearly all patients with neuropathic ADM findings, only a minority of genetic findings were clearly linked to enteric nervous system disease. The often nonspecific nature of histopathological findings in both myopathic and neuropathic PIPO, as observed in our cohort, continues to pose challenges for gaining mechanistic insights from tissue biopsies. Current diagnostic modalities, while informative, are often insufficient to fully elucidate the complex etiologies of neuropathic PIPO.

The observed phenotypic variability, even among people with the same *ACTG2* mutation, highlights the potential influence of genetic, environmental, or epigenetic factors on disease severity and clinical manifestations. If these disease modifiers could be identified, new treatment approaches may ensue.

Our findings underscore that further studies evaluating the mechanistic etiologies of PIPO are needed. Ultimately, a clearer understanding of these diverse mechanisms will pave the way for more precise diagnostic criteria, identification of novel therapeutic targets, and development of personalized treatment strategies to improve outcomes for children suffering from this devastating condition.

## CONFLICT OF INTEREST STATEMENT

Hayat Mousa is on the Scientific advisory board for the World Visceral Myopathy Foundation Robert O. Heuckeroth is on the Scientific Advisory Board for Neurenati Therapeutics and the World Visceral Myopathy Foundation, and has served on Scientific Advisory Boards for Millenium Pharmaceuticals (Takeda) and as a consultant for BlueRock Therapeutics.
